# Mechanistic connotations of restriction of intramolecular motions (RIM)

**DOI:** 10.1093/nsr/nwaa260

**Published:** 2021-06-11

**Authors:** Yujie Tu, Zheng Zhao, Jacky W Y Lam, Ben Zhong Tang

**Affiliations:** Department of Chemistry, Hong Kong Branch of Chinese National Engineering Research Center for Tissue Restoration and Reconstruction, Institute for Advanced Study and Department of Chemical and Biological Engineering, The Hong Kong University of Science and Technology, China; Department of Chemistry, Hong Kong Branch of Chinese National Engineering Research Center for Tissue Restoration and Reconstruction, Institute for Advanced Study and Department of Chemical and Biological Engineering, The Hong Kong University of Science and Technology, China; Department of Chemistry, Hong Kong Branch of Chinese National Engineering Research Center for Tissue Restoration and Reconstruction, Institute for Advanced Study and Department of Chemical and Biological Engineering, The Hong Kong University of Science and Technology, China; Department of Chemistry, Hong Kong Branch of Chinese National Engineering Research Center for Tissue Restoration and Reconstruction, Institute for Advanced Study and Department of Chemical and Biological Engineering, The Hong Kong University of Science and Technology, China; Center for Aggregation-Induced Emission, SCUT-HKUST Joint Research Institute, State Key Laboratory of Luminescent Materials and Devices, South China University of Technology, China; AIE Institute, Guangzhou Development District, China; 4 Ming Wai Lau Centre for Reparative Medicine, Karolinska Institutet, China

## Abstract

Restriction of intramolecular motion (RIM) is the widely-accpeted mechanism of aggregation-induced emission (AIE). In this concise and comprehensive perspective, four mechanistic models related to different nonradiative pathways are summarized with examples to disclose the connotation of RIM, and meaningful mechanistic topics for future researches are advised.

Molecules are all in constant motions, which influence their properties. For example, in photophysics, the light emission behavior of a luminogen is determined by its electronic and nuclear motions in the excited state. Flexible molecular motions usually favor nonradiative decay along with the transformation of excited-state energy to other forms such as thermal energy. Therefore, the restriction of intramolecular motions (RIM) is commonly adopted to achieve luminescent materials with high emission brightness.

Historically, scientists often believed that the properties of a substance are determined by the properties of a molecule. Thus, early research into luminescent materials focused mainly on the properties of isolated molecules in dilute solution. Accordingly, the strategy of RIM relies on structural rigidification at the molecular level such as introducing fused aromatic rings. However, in reality, novel properties that molecules may not have, may emerge in aggregates. For example, 1) hydrophilic amino acids can form hydrophobic proteins in hierarchical structures, and 2) some nonconjugated molecules, such as sugar, can emit light when clustered in a compact aggregate. Therefore, there is no need to only rely on RIM by designing molecules with rigid structures. Instead, we can suppress molecular motions at the mesoscopic level [1,2].

The exploration into aggregation-induced emission (AIE) is good practice to achieve turn-on luminescence by RIM at the aggregate level. Scientists have attempted to design AIE luminogens (AIEgens) with rotors/vibrators which are non-emissive in the solution state but highly emissive in the aggregate state through restriction of intramolecular rotation/vibration (RIR/RIV) (Fig. 1A) [1]. However, not all motions cause luminescence quenching. In recent years, numerous studies have been conducted to identify the critical molecular motions responsible for nonradiative transitions and elucidate the excited-state deactivation pathways of AIEgens from the quantum-chemical perspective. Different models have been established to disclose the connotation of the RIM mechanism (Fig. 1B).

**Figure 1.**
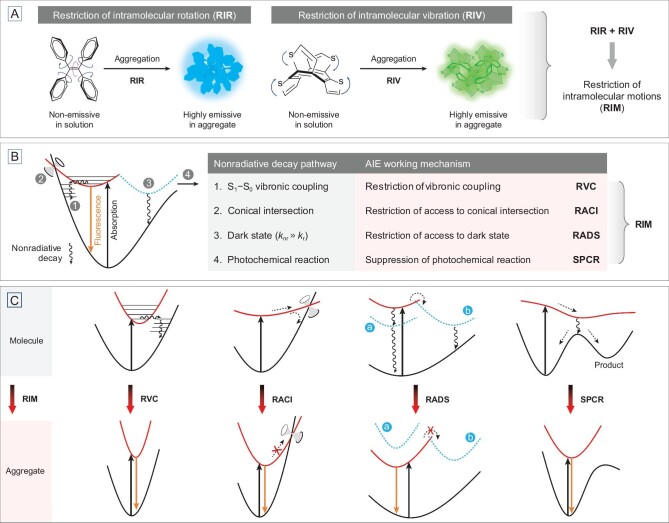
(A) Working mechanism of aggregation-induced emission (AIE): restriction of intramolecular motions (RIM) including rotation (RIR) and/or vibration (RIV). Adapted with permission from Ref. [1], Copyright Wiley-VCH. (B) Activation of RIM through blocking various nonradiative pathways. *k*_r_ = radiative decay constant, *k*_nr_ = nonradiative decay constant. (C) Potential energy surfaces for the nonradiative and radiative pathways at molecular and aggregate levels, respectively.

First, for AIE systems with active molecular motions, the internal conversion caused by S_1_–S_0_ vibronic coupling is often very fast to surpass the fluorescence. For example, AIEgen **1** undergoes phenyl-ring torsion and double-bond twisting upon excitation (Fig. 2A), which allows strong vibronic interactions between S_1_ and S_0_ [3]. However, because of RIM in aggregates, its potential energy surfaces (PESs) become sharp and steep. A small scale of nuclear displacement may give rise to a big potential energy elevation. In the aggregate state, there are fewer vibrational modes in S_1_ and S_0_, and overlap of their wavefunctions is less effective [4]. Thus, the AIEgens are emissive in the aggregate state because of restriction of the S_1_–S_0_ vibronic coupling (RVC) (Fig. 1C).

Second, many AIE molecules in the excited state undergo flexible molecular motions and rapidly relax to a conical intersection (CI) where the S_1_ and S_0_ are degenerate, the magnitude of vibronic interactions approaches infinity, and the exciton decays nonradiatively. However, the molecular motions that lead to the CI geometry, such as the molecular motions of AIEgen **2**/**3**/**4** indicated in Fig. 2B, can be restricted upon aggregation [5–7]. The emission is restored by restriction of access to the conical intersection (RACI) (Fig. 1C).

Third, excited states have different characteristics because of differences in terms of the transition origin (e.g. (π, π^*^), (n, π^*^), (n,$\ {\rm{\sigma }}$^*^), (π,$\ {\rm{\sigma }}$^*^)), the spatial overlap of transition orbitals (e.g. locally excited (LE) or charge transfer (CT)), the state of multiplicity (e.g. singlet or triplet) and the symmetry of transition (e.g. symmetry-allowed or symmetry-forbidden). Some excited states exhibit small molar absorptivity and oscillator strength, thus consequently lead to a low transition probability and a much larger nonradiative decay constant than the radiative decay one (*k*_nr_$ \gg $*k*_r_). These kinds of excited states favor the nonradiative decay thus defined as dark states. The (n, π^*^) state/CT state/triplet state/symmetry-forbidden transition are dark states for fluorescence when compared to (π, π^*^) state/LE state/singlet state/symmetry-allowed transition, respectively. Taking heteroatom-containing AIEgens **5**/**6**/**7** as examples, their weak fluorescences in the solution state are ascribed to photo-induced electron transfer (PET), twisted intramolecular charge transfer (TICT) and intersystem crossing (ISC), respectively [8–10]. In fact, these photophysical processes can be unified as the quenching effect of (n, π^*^) dark states. The twisting of lone pair-bearing moieties can modulate the overlap between n orbital and π plane to result in the n–π orbital ordering reversal and the transformation of the (π, π^*^) bright state to the (n, π^*^) dark state (Fig. 2C). However, in the aggregate state, the molecular motions that lead to the dark state are restricted or the energy of the dark state is raised, which makes the dark state kinetically or thermodynamically inaccessible. Therefore, the emission is recovered as a result of restriction of access to the dark state (RADS) (Fig. 1C).

Besides the photophysical decay pathways, the excited-state AIEgens may undergo photochemical reactions such as photoisomerization and photocyclization (Fig. 2D). Upon excitation, AIEgens such as **8** and **9** undergo conformational changes along the reaction coordinate to a ‘watershed’ between the reactant and the product, where the nonradiative decay is dominant because of either the strong vibronic coupling or the presence of a conical intersection [11,12]. Meanwhile, the new products formed are possibly non-emissive based on their own photophysical properties. However, in the aggregates, the emission is turned on by suppression of the photochemical reaction (SPCR) by restricting the molecular motions that lead to product formation (Fig. 1C).

Above all, in this concise perspective, four mechanistic models related to different nonradiative pathways have been summarized with schematic illustrations and straightforward examples to disclose the connotation of the RIM mechanism. In future research, besides revealing the deactivation pathways and identifying the exact molecular motions that account for luminescence quenching, other mechanistic topics regarding molecular motions are also worth exploring, including the solid-state molecular motion, the intermolecular translational motions, the frequency and amplitude of molecular motions, etc. Meanwhile, clearer and more comprehensive mechanistic explanations are required on AIE systems with clusterization-triggered emission, room temperature phosphorescence, and so on. Hopefully, by gradually completing the AIE mechanistic picture, we can gain a better understanding of the science in mesoscopic aggregates and achieve novel and diverse AIE materials with intriguing applications.

## Figures and Tables

**Figure 2.**
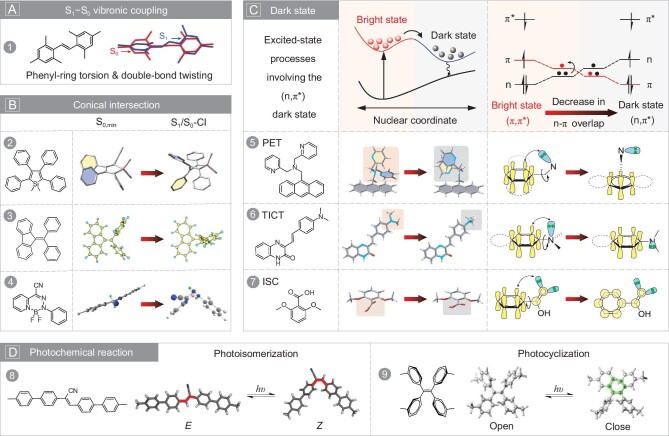
Examples of excited-state molecular motions leading to different nonradiative pathways including (A) S_1_–S_0_ vibronic coupling (adapted with permission from Ref. [3], Copyright Royal Society of Chemistry), (B) conical intersection (adapted with permission from Ref. [5,6], Copyright Royal Society of Chemistry and Ref. [7], Copyright, American Chemical Society), (C) dark state (adapted with permission from Ref. [8,9], Copyright Wiley-VCH and Ref. [10], Copyright ChemRxiv) and (D) photochemical reaction (adapted with permission from Ref. [11,12], Copyright American Chemical Society; further permissions related to Ref. [12] should be related to the American Chemical Society). CI = conical intersection, PET = photoinduced electron transfer, TICT = twisted intramolecular charge transfer, ISC = intersystem crossing.
